# Effect of Laser Therapy on Postoperative Pain and Endodontic Retreatment: A Systematic Review and Meta-Analysis

**DOI:** 10.1016/j.identj.2023.10.012

**Published:** 2023-11-18

**Authors:** Sai Vakul Toopalle, Indu Yadav, Alpa Gupta, Nishant Chauhan, Dax Abraham, Arundeep Singh, Mrinalini, Mudit Sharma, Serena lalfakwami

**Affiliations:** a Department of Conservative Dentistry and Endodontics, Manav Rachna Dental College, Faridabad, Haryana, India; b Boston University Goldman School Of Dental Medicine , Boston, USA

**Keywords:** Photobiomodulation therapy, Postoperative pain, Root canal re-treatment, Low-level laser therapy

## Abstract

**Background:**

Root canal re-treatment (RCR) cases are considered some of the most challenging cases in the field of endodontics, as they are mostly associated with various iatrogenic errors such as ledge formation, incomplete biomechanical preparation, file separation, and incomplete obturation. These iatrogenic errors lead to defective niches within root canals that may act as reservoirs for various viable microorganisms. Such residual microbial niches may cause postoperative pain even after thorough debridement and reshaping the canals, ultimately leading to a poor prognosis for the tooth. Nowadays, prevention of postoperative pain in re-treatment cases and prognosis are effectively managed by photobiomodulation therapy (PBMT).

**Method:**

Relevant studies in the English language published before November 2022 were identified using electronic databases like PubMed, SCOPUS, and EBSCO to conduct bibliographic research. This systematic review is based on 3 studies that were found eligible as per the inclusion and exclusion criteria. This systematic review is in accordance with PRISMA guidelines.

**Results:**

The systematic review indicated a positive impact by significantly decreasing postoperative pain in RCR cases when treated with PBMT. The variation was statistically significant at 24 hours (*P* = .0002), 48 hours (*P* = .03), and 72 hours (*P* = .02). The mean difference at 24 hours was 0.65 (95% CI, 0.32–0.99), at 48 hours was 0.46 (95% CI, 0.05–0.87), and at 72 hours was 0.40 (95% CI, 0.07–0.74). There was no statistical heterogenicity at 24 hours (*P* > .05), but a medium heterogenicity was observed at 48 hours and 72 hours.

**Practical implication:**

PBMT or low-level laser therapy has shown superior results as compared to the conventional pharmacologic approach in postoperative pain management in RCR cases.

## Introduction

Postoperative pain after endodontic treatment continues to be a problem of major clinical issue. The condition not only causes chronic patient discomfort but may potentially trigger emergency department visits. According to Sathorn et al,^1^ 3% to 58% of patients experience pain after endodontic therapy. This is due to the release of inflammatory mediators whenever the pulp or periradicular tissues are chemically, mechanically, or microbially injured during root canal therapy or re-treatment. These mediators trigger central and peripheral hyperalgesia pathways by activating sensitive nociceptors, which causes postoperative pain or discomfort in patients.^2^^,^^3^

Root canal re-treatment (RCR) is advised when posttreatment pain recurs or manifests after root canal therapy. It is thought to eliminate the prime reasons for treatment relapse: primary resistant or secondary invasive bacteria. However, several studies reported pain after RCR in 1% to 16% of the cases.^4^

Pharmacologic and nonpharmacologic techniques are often used to reduce the intensity of endodontic postoperative pain. Pharmacologic methods include prescribing medications like acetaminophen, antihistamines, nonsteroidal or steroidal anti-inflammatory drugs, salicylic acid, narcotic analgaesics, intracanal drugs, or long-acting anesthesia for postoperative pain management. Nonpharmacologic methods include intracanal cryotherapy, various root canal kinematics, intracanal laser irradiation, and low-level laser therapy (LLLT) to reduce postoperative discomfort.^5^ Amongst these techniques, pharmacologic techniques have relatively greater side effects; thus, nonpharmacologic techniques are considered a safe and effective form of postoperative pain reduction.^6^

Mester discovered LLLT in 1967 and defined it as a nonthermal, near-infrared laser with a wavelength of 600 to 1000 nm and an energy output of 5 to 500 mW.^7^^,^^8^ LLLT has demonstrated promising results in dental treatments such as orthodontic discomfort management, symptomatic oral lichen planus treatment, maxillofacial abnormalities repair, and stomatitis prevention.^9^^,^^10^ LLLT is extensively used in endodontic therapy because it helps with wound healing, root canal cleaning, and postoperative pain relief.^11^^,^^12^

In endodontics a photosensitiser (PS) is placed inside the root canal and exposed to LLLT for a specified incubation period. The wavelength used should coincide with the maximum absorption band of the specific photosensitiser in the cananl system. This results in a reaction product of a singlet oxygen (^1^O2) and reactive oxygen species (ROS), which eradicate the microbial cells. Benefits of this approach include wide spectrum of bactericidal activity, the absence of photo-resistant species even after repeated administrations, and minimal host tissue injury. Furthermore, the outcome of the treatment is the same for the antibiotic resistance microorganisms as well.^13^ LLLT applied in endodontics entail using different laser types with wavelengths ranging from 810 nm to 2940 nm.

Indeed LLLT in combination with photobiomodulation therapy (PBMT) is one of the most effective nonpharmacologic techniques that reduces pain. Endorphin production stimulation leading to pain mitigation are thought to be the possible mechanisms that reduce postoperative pain after LLLT.^14^^,^^15^ According to some studies, the analgaesia is due to the anti-inflammatory and neurologic actions of LLLT, which include enhancing lymphocyte and nerve cell respiration, stabilising membrane potentials, and releasing neurotransmitters into the inflamed tissue.^16^ Furthermore, LLLT significantly upregulates fibroblast activity at the tissue level, speeds up connective tissue healing, and has anti-inflammatory effects.^17^^,^^18^ In prior studies, LLLT and intracanal laser irradiation^19^ have demonstrated significant reduction in postoperative endodontic discomfort.^20^

Over the past 2 years, an avalanche of studies and research on the analgaesic effects of LLLT to manage RCR has been reported. However, the efficacy of LLLT for postoperative pain control in RCR has not been reviewed, thus far. Hence, this systematic review aims to summarise the existing data from randomised controlled trials to evaluate the effectiveness of LLLT on postoperative pain after RCR.

## Methods

### Review guidelines and registration

Two reviewers searched the International Prospective Register of Systematic Reviews (PROSPERO) for the listed protocols to find whether there was any review registered which assessed the effect of PBMT on postoperative pain in RCR patients. The systematic review is performed in accordance with Preferred Reporting Items for Systematic reviews and Meta-Analysis (PRISMA) guidelines.^7^

### Eligibility criteria

The selection of the study was done using PICOS elements. In PICOS, Population (P) represents the patients with RCR tooth, Intervention (I) is the PBMT or LLLT, Comparison (C) is with a control group of placebo insisted LLLT or mock laser, Outcome (O) is postoperative pain, and (S) Studies were those that assessed the postoperative in RCR teeth. Articles published up until November 2022 were taken into consideration. However, studies conducted in any language other than English were not included. In addition to this, all the systematic reviews or narratives, letters to the editor, case/reports, opinion articles, experimental research, and conference abstracts were also excluded. Finally, the research question for this systematic review was formulated as follows: “Does LLLT reduce postoperative pain after root canal re-treatment teeth (RCR)?”

### Information sources and literature search strategy

By using a combination of keywords and indexing vocabulary, the Medline database of the US National Library of Medicine (MeSH terms) was searched with an advanced electronic search method. The following terms and subject headings were used in the literature search: photo-biomodulation therapy, low level laser therapy, postoperative pain, and root canal re-treatment teeth. The search approach remained consistent across both Scopus and EBSCO hosts, supplemented by a manual search of grey literature. Two reviewers independently conducted an exhaustive search for relevant studies released up until November 2022, utilising the Boolean operators “OR” and “AND” within the search string (Table 1). After screening 23 records, 8 of them were excluded and only 15 reports were sought for retrieval, amongst which 14 were assessed for eligibility, with only 3 studies ultimately incorporated in the final review. Since only a few articles met the inclusion criteria and only three studies were extracted, neither reviewer disagreed with the search strategy.

**Table 1:** Search strategy applied to the current review.

DATABASE	SEARCH STRATEGY	N
PUBMED	(endodontic retreatment OR root canal retreatment) AND (pain OR discomfort OR analgesia) AND (laser OR laser therapy OR laser irradiation OR phototherapy OR low-level laser OR low-intensity laser OR low-output laser OR soft laser)	**15**
SCOPUS	(endodontic retreatment OR root canal retreatment) AND (pain OR discomfort OR analgesia) AND (laser OR laser therapy OR laser irradiation OR phototherapy OR low-level laser OR low-intensity laser OR low-output laser OR soft laser)	**9**
EBSCO HOST	(endodontic retreatment OR root canal retreatment) AND (pain OR discomfort OR analgesia) AND (laser OR laser therapy OR laser irradiation OR phototherapy OR low-level laser OR low-intensity laser OR low-output laser OR soft laser)	**753**

**Fig. 1:**
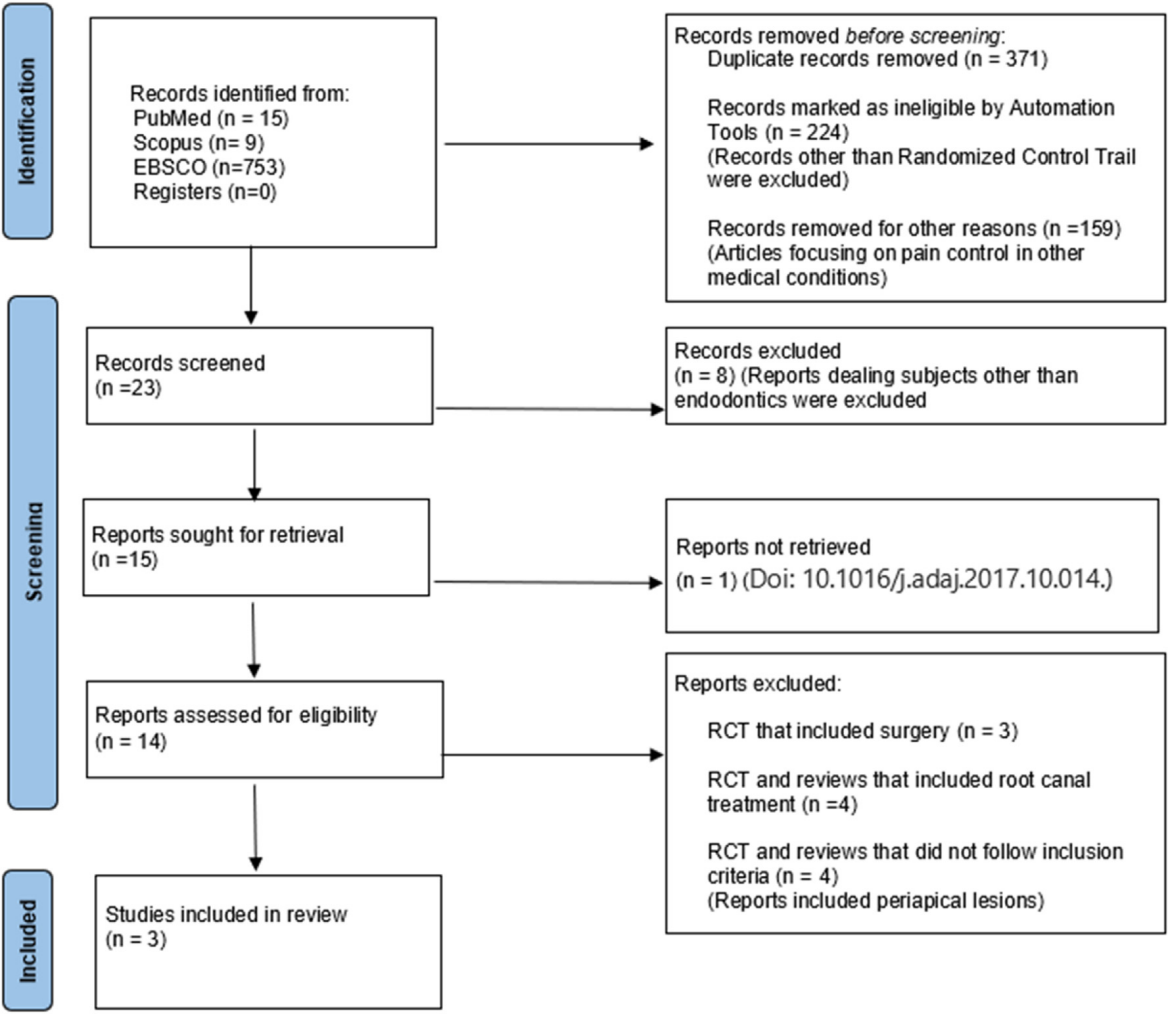
PRISMA flowchart depicting the workflow of the review.

The steps undertaken during the selection process are highlighted in Figure 1.

### Data extraction and quality assessment

The data were extracted and collected in a standard format, as shown in Table 2, which included: the authors, number of patients based on gender, diagnosis, tooth type, tooth number associated with each group, number of dropout participants, type of endodontic treatment, evaluation method, evaluation time interval, and observed outcomes. Necessary efforts were made to gather the required data by contacting the authors of relevant studies when the data were unclear or unavailable. Researchers rated studies with 1 to 3 missing items as low-risk, 4 to 6 missing items as having moderate bias, and those with more than 6 missing elements were rated as high-risk.^21^

**Table 2:** Study characteristics of individual studies.

Study ID	No. (M/F)	Diagnosis	Tooth type	Groups	No. of drop out	Endodontic treatment	Evaluation method	Evaluation interval/considered interval	Observed outcomes
Hakan Arslan	PLD-13/4 LD-8/8	Periapical lesions with scores 2 or 3 in accordance with the periapical index	Symptomatic mandibular molars	LLLT-16 MOCK LASER-17	2 from LLLT 1 from placebo	2 visits using intracanal medicament	VAS scale	4, 8, 12, 24, and 48 hours 24 hours, 48 hours, 72 hours	24 hours (*P* < .05) Significant 48 hours (*P* < .05) Significant 72 hours (*P* < .05) Significant
Ozgur Genc Sen	PLD-15/21 LD-19/18	Chronic periapical infection (minimum size of lesion, 2*2 mm	Single-rooted teeth	LLLT-42 MOCK LASER-42	2 in each group	A single visit	NRS scale	24, 48, and 72 hours 24 hours, 48 hours, 72 hours	48 hours showed significantly less pain in LD group than in PLD group (*P* < .05) and 72 hours showed insignificant difference (*P* > .05)
Mahta Fazlyab	NR	Chronic periapical infection	Symptomatic first or second mandibular molars	LLLT-18 MOCK LASER-18	0	A single visit	VAS scale	4, 8, 12, and 24 hours and 2, 3, and 7days 24 hours, 48 hours, 72 hours	Both 24 hours and 48 hours showed intense pain in both groups with [mean (SD) = 0.22 (0.54)]

LD, laser disinfection; PLD, pseudo laser disinfection; VAS scale, visual analogue scale; NRS scale, numerical rating scale.

## Results

Upon conducting a comprehensive literature search, 777 articles were initially retrieved from various databases. After removing the duplicates, only 15 studies remained. The full text of these 15 articles was retrieved and assessed by screening the titles and abstracts. Finally, only 3 studies were included in the systematic review for qualitative analysis (Figure 1). The included studies were carefully examined to identify similarities, and a meta-analysis was performed. Comparable results were combined using the RevMan 5.3 software program for quantitative data synthesis. The studies provided mean and standard deviation data on postoperative pain at 24 hours, 48 hours, and 72 hours, which were extracted for analysis.

The effect size variable of data was determined using the mean difference method. In order to establish a comparable index across all studies, the mean difference in each study was divided by the standard deviation, resulting in a standardised mean difference (SMD). SMD was computed for each study. The statistical heterogeneity between studies was analysed using the *I*^2^ value, which indicated low, medium, and high heterogeneity at 25%, 50%, and 75%, respectively.

### Quantitative analysis

**Fig. 2:**
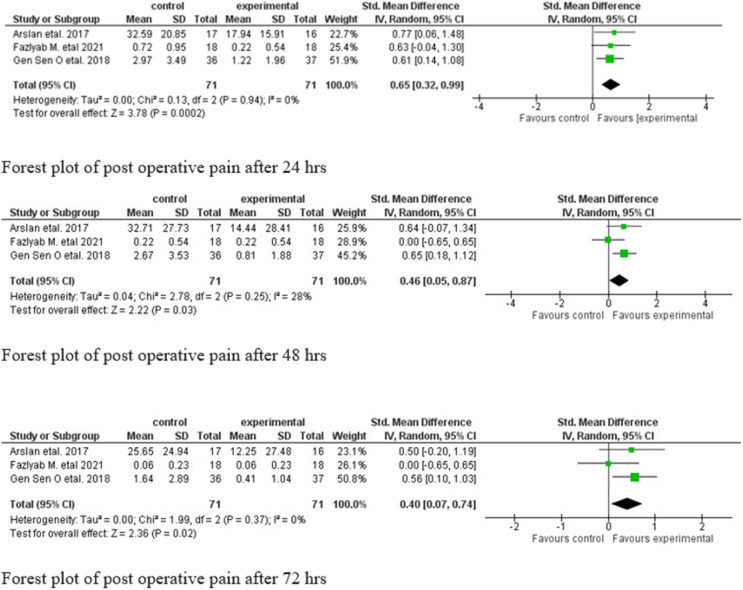
Forest plot of post operative pain after 24, 48, and 72 hours.

Figure 2 shows Forest plot illustrations of effect of LLLT and pseudo LLLT on postoperative pain in 24 hours, 48 hours, and 72 hours. In current analysis, the standard mean difference obtained at the mean difference at 24 hours was 0.65 (95% CI, 0.32–0.99), at 48 hours was 0.46 (95% CI, 0.05–0.87), and at 72 hours was 0.40 (95% CI, 0.07–0.74). The statistical variation was significant at 24 hours (*P* = .0002) and at 48 (*P* = .03). and 72 hours (*P* = .02). There was no statistical heterogenicity at 24 hours with *I*^2^ = 0% (*P* > .05) and medium heterogenicity at both 48 hours and 72 hours.

### Study characteristics

All 3 selected studies were published between 2017 and 2021, with one each in 2017, 2019, and 2021.

Out of 3 studies, 2 used visual analogue score, while the third used numeric rating scale to evaluate pain. Additionally, 2 of the studies considered the periapical index score. Regarding RCR, 2 studies opted for a single-visit procedure, while the third chose a 2-visit approach. Diode laser with different wavelengths ranging from 940 nm to 980 nm were used in all the studies. Diode lasers usually deliver wavelengths ranging from 810 to 1064 nm. However, the wavelength was limited to 940 to 970 nm for this review. Amongst the 3 studies, Genc Sen et al^22^ used 940 nm, Arslan et al^23^ used 970 nm, and Fazlyab^24^ used 980 nm wavelength for LLLT to obtain low levels of postoperative pain. Another factor that influences LLLT is the mode of emission, which refers to method of laser irradiation (ie, whether it is continuous mode or pulse wave mode). According to various references and case studies,^25^, ^26^, ^27^ pulse wave mode is more advantageous than continuous wave mode because pulsing wave or pulsing regime has better dissipation of heat. Amongst the 3 studies, Genc Sen et al^22^ used a continuous mode of emission and the other 2 studies ^23^^,^^24^ did not specify the mode of emission.

Additionally, this analysis highlighted the fact that there was some discrepancy in the exposure times of the selected studies. For instance, a study by Arslan^23^ used LLLT for a maximum of 30 seconds per tooth, whereas Fazlyab^24^ only exposed for 15 seconds per tooth. Meanwhile, Genc Sen et al^22^ did not provide any information on the duration of exposure.

Moreover, the fiber tip diameter used in LLLT also varied between studies, as 2 studies utilised a diameter of 220 µM^(^^23^^,^^22^^)^ while one study did not specify the fiber tip diameter.^24^ Two studies used laser by irradiating buccal and lingual mucosa over the root apices. In contrast, the other study used laser irradiation at the root tip by gaining access through the root canal. Pain levels were assessed at 24 hours, 48 hours, and 72 hours postirradiation with LLLT, which was the standard procedure for all 3 studies included in this review.

### Assessment of risk of bias

Table 3 shows the results of the evaluation of the risk of bias amongst all the 3 studies included in this review ranges from very low to low risk of bias.^28^

**Table 3:** Assessment of risk of bias.

Critical appraisal tools for use in JBI Systematic Reviews	Hakan Arslan	Ozgur Genc Sen	Mahta Fazlyab
Was true randomisation used for assignment of participants to treatment groups?	Yes	Yes	Yes
Was allocation to treatment groups concealed?	Yes	Yes	Yes
Were treatment groups similar at the baseline?	Yes	Yes	Yes
Were participants blind to treatment assignment?	Yes	Yes	Yes
Were those delivering treatment blind to treatment assignment?	Yes	Yes	Yes
Were outcomes assessors blind to treatment assignment?	Yes	Unclear	Yes
Were treatment groups treated identically other than the intervention of interest?	Yes	Yes	Yes
Was follow-up complete and, if not, were differences between groups in terms of their follow-up adequately described and analysed?	Yes	Yes	Yes
Were participants analysed in the groups to which they were randomised?	Yes	Yes	Yes
Were outcomes measured in the same way for treatment groups?	Yes	Yes	Yes
Were outcomes measured in a reliable way?	Yes	Yes	Yes
Was appropriate statistical analysis used?	Yes	Yes	Yes
Was the trial design appropriate and any deviations from the standard RCT design (individual randomisation, parallel groups) accounted for in the conduct and analysis of the trial?	Yes	Yes	Yes

## Discussion

This study aimed to evaluate the effectiveness of LLLT in managing postoperative pain in RCR cases. Our systematic review and meta-analysis comprised 3 studies that focussed on RCR cases.

PBMT is a noninvasive technique that uses photonic energy at specific wavelengths ranging from 600 to 1100 nm to induce a biological response and modulate biological processes within that tissue through energy transfer.^21^ This therapy elicits anti-inflammatory, analgaesic, and therapeutic action with a correct incident dose. According to a study, PBMT does not have any discernible thermal effects on irradiated tissues.^22^ Additionally, various in vivo studies report that PBMT impedes nerve function through local conduction blockade, axonal flow interruption, targeted nociceptor inhibition, and other changes.^29^ These alterations reduce discomfort and are reversible without adversely impacting the patient's general health. Therefore, LLLT is a reliable nonpharmacologic approach to pain management that follows the principle of PBMT.

LLLT has been applied in endodontics using different lasers with wavelengths ranging from 810 nm to 2940 nm and has demonstrated a significant reduction in postoperative pain after a single-visit root canal treatment (RCT).^30^^,^^31^ Similar results were observed when LLLT was used after endodontic surgery.^32^^,^^33^ From 2011 to 2021, 8 studies were published, which were contemporaneous trials, with 5 of them evaluating the use of LLLT in pain management following RCT,^30^^,^^34^, ^35^, ^36^ while the remaining 3 investigated the role of LLLT in pain management following RCR. Amongst variety of and the remaining 3 investigating the role of LLLT in pain management following RCR. This systematic review has included studies that perform LLLT using the DIODE laser since it has advantages like high power output, ease of manufacturing, cost-effectiveness, greater efficiency, smaller size, and ease of application.^37^

Although various factors impact the outcome of LLLT, including wavelength, mode of emission, power, fiber tip of the laser, time, and method of application, this review found no standardised wavelength, mode of emission, power, or fiber tip amongst the selected studies. All 3 studies showed promising significant results, and further research should focus on these parameters to enhance the outcome levels.

LLLT requires precise timing for effective results, as an ambiguous time frame can lead to ineffective laser exposure. A very short application time may not affect the pathology, whereas applications with excessive time may cause be deleterious causing tissue damage. Amongst the studies mentioned in this review, a variation in the exposure time was observed, with one study exposing LLLT for up to 30 seconds per tooth^23^ and another study exposing it for 15 seconds per tooth.^24^ In contrast, one study did not specify the exposure time.^22^ However, the time used for LLLT independently depends upon energy output, which is related to the heat tolerance of the tissue. Further studies should consider and standardisation of the time duration.

Another factor to be taken into consideration is the method of application of the laser during the time of exposure which in turn influences the outcome of LLLT. In endodontics, 2 basic exposure methods are used: (1) inserting the probe tip of the laser into the root canal and inducing LLLT and (2) exposing the laser to the mucosa over the apices of the target tooth. Amongst the 3 studies included in this systematic review, 2 studies exposed the laser on the buccal and lingual mucosa apices^23^^,^^24^ and one study by inserting the laser tip in to the root canal.^22^ A recent study found that lingual and buccal irradiation was considerably more efficient than buccal-only irradiation for treating postoperative endodontic pain 8 hours later.^38^ However, no rationale was given for the latter methods of application and its benefits.

Further consideration in the factors that influences the outcome of LLT should be the number of visits during RCR. Two of the 3 selected studies did not use intracanal medicament, whereas a study by Harklan et al^31^ performed 2-visit RCR, with the placement of calcium hydroxide as an intracanal medicament between appointments.

The limitations of this review are that all 3 studies included had some variations regarding parameters followed during the process of LLLT. However, all 3 studies have shown significant results favouring the experimental group compared to the control group, as depicted in the Forest plot. This clearly indicates that there are positive significant results of LLLT in RCR after 24 hours, 48 hours, and 72 hours, respectively.

## Conclusion

The available literature indicates that LLLT can effectively alleviate postoperative pain and improve patient satisfaction in RCR cases. This reduction in pain demonstrates the effectiveness of LLLT as it reduces the inflammation and microbial load significantly, which is the prime goal of a successful endodontic treatment. However, since the studies included in this systematic review were limited to only 3 and had varying laser characteristics, it is difficult to determine a single parameter that would effectively indicate the best results in postoperative pain reduction using LLLT. Therefore, future research should focus on utilising standardised protocols and improved methods to optimise pain management techniques using LLLT.

## Conflict of interest

None disclosed.
